# A Low-Cost Time-Correlated Single Photon Counting Portable DNA Analyzer

**DOI:** 10.3390/s19132838

**Published:** 2019-06-26

**Authors:** Yi Tian, Liping Wei, Derek Ho

**Affiliations:** Department of Materials Science and Engineering, City University of Hong Kong, Tat Chee Avenue, Kowloon 999077, Hong Kong, China

**Keywords:** photon-counting, TCSPC, DNA detection, liquid-core waveguide

## Abstract

Photon-counting analysis of nucleic acids plays a key role in many diagnostics applications for its accurate and non-invasive nature. However, conventional photon-counting instrumentations are bulky and expensive due to the use of conventional optics and a lack of optimization of electronics. In this paper, we present a portable, low-cost time-correlated single photon-counting (TCSPC) analysis system for DNA detection. Both optical and electronic subsystems are carefully designed to provide effective emission filtering and size reduction, delivering good DNA detection and fluorescence lifetime extraction performance. DNA detection has been verified by fluorescence lifetime measurements of a V-carbazole conjugated fluorophore lifetime bioassay. The time-to-digital module of the proposed TCSPC system achieves a full width at half maximum (FWHM) timing resolution from 121 to 145 ps and a differential non-linearity (DNL) between −8.5% and +9.7% of the least significant bit (LSB) within the 500 ns full-scale range (FSR). With a detection limit of 6.25 nM and a dynamic range of 6.8 ns, the proposed TCSPC system demonstrates the enabling technology for rapid, point-of-care DNA diagnostics.

## 1. Introduction

Nucleic acids, with deoxyribonucleic acid (DNA) being the most prominent example, provide substantial diagnostic information for infectious diseases, genetic disorders, cancer mutations, and fetal abnormality. DNA sequences are routinely used as controls for clinical genomics [1], biomarkers for cancer [2], and biophysical analytical tools to quantify the effect of drugs [3].

As one of the well-adopted techniques in medical diagnostics and biological research of nucleic acids, fluorescence spectroscopy has attracted much attention recently. Optically-mediated DNA detection is based on identifying small changes in light emitted from typically fluorescent molecules as labels to the nucleic acid, with numerous examples in the recent literature. A method for the fluorescence detection of the cellular mismatch repair ability in human cells is proposed to improve the detection of repair defects [4]. A fluorescence-coded DNA nanostructure probe system is presented to enable discrimination of tumor heterogeneity via a screening of dual intracellular microRNA signatures in situ [5].

The fluorescence spectroscopy for DNA analysis can be broadly classified into two categories: Steady-state and time-resolved. Steady-state analysis involves constant illumination of the sample with a continuous beam of light while the emission spectrum is recorded by scanning the emission monochromator and corrected for detector response. Although the instrumentation hardware requirement is lower, steady-state techniques typically observe the average of the phenomenon, thus unable to capture dynamics of the biological processes. In contrast, time-resolved analysis is performed with a pulsed light source, and the decay of the fluorescence intensity is acquired as a function of time, using fast detection systems [6].

As a prominent time-resolved technique, time-correlated single photon counting (TCSPC) has gained extensive interest due to its short measurement time, high sensitivity, high counting efficiency, high timing resolution, and high signal-to-noise ratio (SNR). TCSPC measurement is based on the repetitive detection of elapsed time from the excitation photon to the corresponding emission photon with timing resolution up to sub-nanoseconds. A histogramming process that records the photon density over the time of the fluorescence decay is constructed. The shape of the histogram represents the fluorescence emission of the excited sample, from which the fluorescence lifetime can be extracted.

A TCSPC system consists of both optical and electronic subsystems, in which the most critical capability is to attenuate excitation light while transmitting emission light (a.k.a., emission filtering). Numerous approaches have been taken to separate the emission light from the excitation light. For example, multiple-layer distributed Bragg reflectors (DBRs) [7] are used to transmit light within a desired spectral range. DBRs can be readily integrated into microscale systems, but variations of a few nanometers in the thicknesses of the layers can cause large errors in the cutoff wavelength, up to ±50 nm. Single-layer absorption filters, whose response is independent of the angle of incidence, are used to absorb excitation light and transmit emission light [8,9]. However, the material of the absorption filter must be specific to the fluorophore, limiting the range of applications. Multi-color sensors are employed to discriminate between excitation light and emission light electronically by exploiting spectral selectivity [10,11]. Although these devices can detect several emission wavelengths without optical filters, spectral resolution is limited, which compromises accuracy. Light-guiding components are used to prevent the excitation light from reaching the detectors due to the collimation of excitation light and isotropy of emission light [12,13]. Attenuation of excitation can be achieved by free-space optical elements without thin film filters, but non-idealities in fabrication can severely affect attenuation efficiency. 

Recently, the liquid-core waveguide (LCW), operating based on total internal reflection (TIR), has gained extensive interests in fluorescence sensing systems as it can avoid strong excitation light interference, and has a high efficiency in collecting emission light [14,15,16]. Many researchers have shown that LCW is applicable to various biomedical applications, such as the detection of DNA, virus, and cells. However, as the systematic design of LCWs for effective emission filtering has yet been well understood, the full potential of LCW has yet been realized.

Conventional photon counting systems for lifetime analysis are typically bulky and expensive, with much prior work having a low level of integration [17,18,19]. Although fluorescence lifetime analytical systems integrated on chip level have also been proposed [20], the fluorescence lifetime analysis (i.e., scientific instrumentation) application is targeting at a low-to-medium volume of production, where implementation of discrete components has the superiority of low startup cost, good flexibility in design revisions, and short production time. Therefore, improvements on fluorescence lifetime analytical systems implemented by discrete components in terms of accuracy, size, and level of integration is of huge demand.

A portable TCSPC analytical system suitable for point-of-care DNA detection is presented in this paper. An LCW guides the excitation to the samples while simultaneously providing emission filtering, all from a single component, leading to the overall system compactness. Experimental results with fluorescence-labelled DNA bioassay show good detection limit and high timing resolution, corroborating the high suitability of the system for portable medical diagnostic applications.

## 2. System Architecture

**Figure 1:**
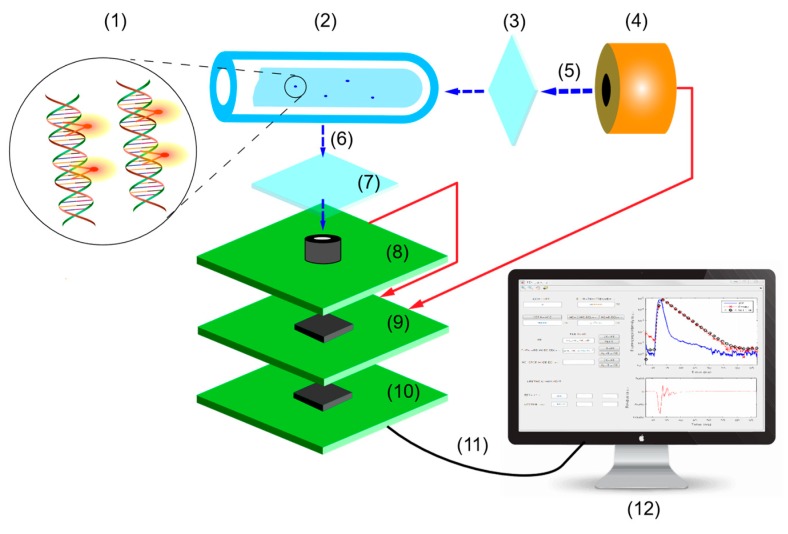
Schematic of the proposed time-correlated single photon-counting (TCSPC) system for DNA analysis. (1) DNA sample bound with fluorophores; (2) liquid-core waveguide (LCW); (3) thin-film neutral density (ND) filter; (4) excitation source; (5) excitation light; (6) emission light; (7) band-pass filter (optional); (8) photon acquisition; (9) time-to-digital conversion; (10) microcontroller unit (MCU); (11) USB connection; (12) data analysis and control.

As depicted in Figure 1, the DNA sample bound with fluorophores is held within the LCW and excited by a pulsed source. Filters can be optionally employed to further improve the excitation attenuation performance, though strictly not necessary. After the emission light reaches the detector, the elapsed time from an excitation photon to the corresponding emission photon is digitized by the time-to-digital conversion module. The digitized results are pre-processed by a microcontroller unit (MCU) before transmitting to a PC. Finally, the histogramming and fluorescence lifetime extraction is performed and displayed. The DNA detection capability of the proposed system is verified by the fluorescence lifetime measurement of highly sensitive V-carbazole fluorophore with and without double-stranded DNA (dsDNA).

## 3. DNA Detection Chemistry

**Figure 2:**
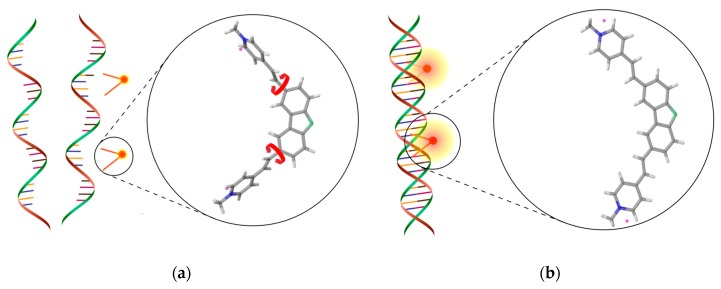
The turn-on phenomenon of the V-carbazole fluorescent molecule. (**a**) OFF state: V-carbazole molecules are free in the solution. The red arrows indicate that the two arms can rotate freely, resulting in a weak fluorescence signal. (**b**) ON state: V-carbazole molecules are bound to dsDNA. The two arms are restricted from rotation, resulting in fluorescence signal enhancement.

DNA detection of the proposed system is realized by the detection of turn-on phenomenon. The turn-on fluorescent molecule is a customized carbazole-based biscyanine with a bischromophoric skeleton, V-carbazole [21]. The molecule has been synthesized to support minor groove binding, which achieves optical switching via rotation of its two arms, is highly sensitive as a probe for dsDNA. As shown in Figure 2, when the V-carbazole molecules are free in the solution, the two arms can rotate freely. The excited electrons dissipate their excess energy rapidly to the solvent non-radiatively, resulting in a weak fluorescence signal, which corresponds to the OFF state. When the V-carbazole molecules are bound with dsDNA, the two arms are restricted from rotation. Therefore, more excited electrons can return to the ground state radiatively, resulting in enhanced fluorescence signal, which corresponds to the ON state. The modulation of fluorescence is utilized to quantify the dsDNA in a given sample.

**Figure 3:**
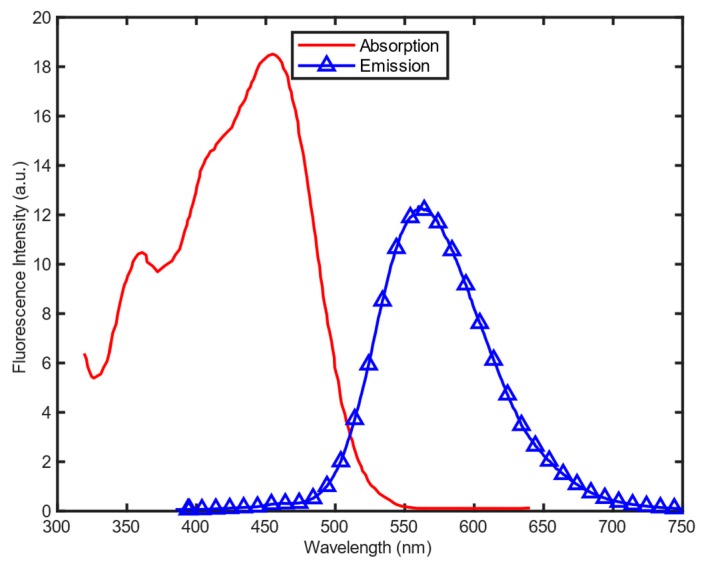
Absorption and emission spectra of V-carbazole.

The absorption and emission spectra of the V-carbazole molecules haven been measured, with results depicted in Figure 3. The number of photons emitted is smaller than the number absorbed, reflecting the existence of non-radiative pathways for the decay of the fluorophore from its excited state. The peak of absorption spectrum is approximately 455 nm, whereas emission spectrum ranges from 475 nm to 675 nm. With a Stokes shift as large as 100 nm, the synthesized V-carbazole relaxes the emission filtering requirement on the optics in the instrumentation.

To provide both minor groove binding accessibility and clinical practicability, the DNA target sequences used for characterization in this work were synthesized to coincide with the sequences of lipid-functionalized DNA nanocages for drug delivery applications [22], using a BioAutomation MerMade MM6 DNA synthesizer with the following nucleobase compositions:

15 DNA: 5′-CTG AGA CTG GAA TGA-3′
15 complimentary DNA: 5′-TCA TTC CAG TCT CAG-3′

DNA synthesis was performed on a 200 nmole scale with 1000 Å nucleotide modified controlled pore glass as solid support. The coupling efficiency of the bases was monitored by trityl bar levels. The DNA strands were de-protected from the solid support by concentrated ammonium hydroxide at 55 °C for 16 h. The crude product was purified by 15% polyacrylamide gel electrophoresis (PAGE). The DNA strands were then mixed in 8 × 10^−5^ M of phosphate buffer with pH 7, subsequently heated to 60 °C and cooled to 4 °C for 4 h by a thermal cycler. To tradeoff between sensitivity and dynamic range in DNA detection, the concentration of V-carbazole was diluted in a pH 7 phosphate buffer solution was chosen to be 6 × 10^−7^ M. Then, V-carbazole at 6 × 10^−5^ M was added to the DNA samples to yield a final concentration of 6 × 10^−7^ M of V-carbazole and a variety of concentrations of DNA sample. The samples were stored at 4 °C for subsequent use.

## 4. System Implementation

**Figure 4:**
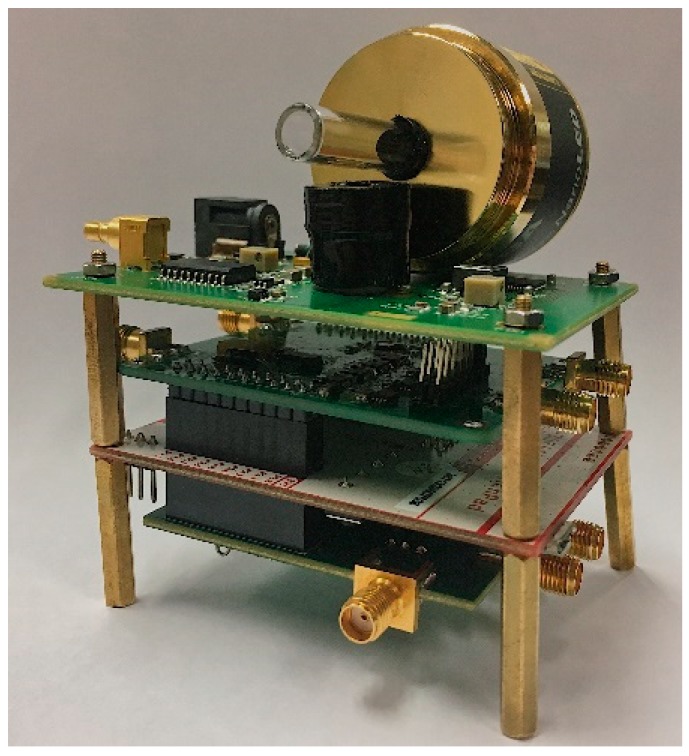
Photograph of the proposed TCSPC system, measuring 7.3 × 6.1 × 9.2 cm^3^.

The implementation of a complete portable TCSPC system for DNA detection requires various optical and electronic components. Each component has been carefully selected to meet the requirements of compactness, excitation rejection, and accuracy. The system has dimensions of 7.3 cm (L) × 6.1 cm (W) × 9.2 cm (H), as shown in Figure 4.

### 4.1. Optics Implementation

#### 4.1.1. Excitation Source

An excitation source, which emits pulsed signal at the absorption wavelength range of the employed fluorescent molecules is required in TCSPC applications. Therefore, to meet the absorption spectrum of V-carbazole depicted in Figure 3, the HORIBA NanoLED N-455 has been selected, with 7 pJ power per pulse, 455 ± 10 nm peak wavelength, and 1.3 ns full width at half maximum (FWHM) pulse width.

#### 4.1.2. Photon Detector

Photomultiplier tubes (PMTs) and single-photon avalanche diodes (SPADs) are the most common photon counting detectors in TCSPC applications to-date. PMTs offer no dead time, large active area (up to cm^2^), and the capability of low dark current recording. However, they are comparatively fragile and bulky (compared to solid-state devices). Although a dead time is followed by each SPAD detection event, SPADs have strong compatibility with CMOS processes, small jitter in pulse response, and strong immunity to electromagnetic interference [23]. Therefore, a CMOS silicon chip that combines an SPAD of 50 μm diameter and less than 50 ns deadtime is employed as the photon detector. With the coverage of spectral range from 350 to 900 nm, ID101-50 is suitable for emission detection of the V-carbazole.

#### 4.1.3. Optical Components

**Figure 5:**
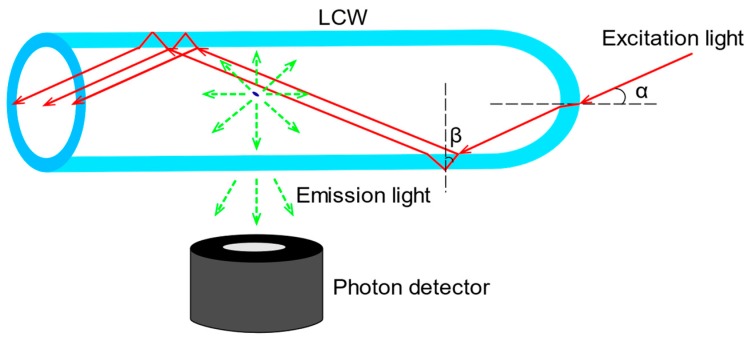
Schematic of the LCW, performing the simultaneous functions of excitation guiding and emission filtering. The red solid lines represent excitation light while the green dashed lines represent emission light. Provided the incident angle α is appropriate, only emission light is allowed to reach the detector.

The selection of sample container is of critical importance in TCSPC applications because it influences both the compactness and optical performance of the system. According to the split-mode 3D ray tracing analysis [14], a quartz LCW with round/round cross-section geometry (see Figure 5) is selected for the proposed system to achieve both sample containment and excitation rejection. As depicted in Figure 5, excitation light emitting into the LCW with an incident angle α, designed to be smaller than the critical angle, is guided within the LCW. As the excitation light reaches the inner side wall of the LCW, partial reflection occurs because the refractive index of the wall is larger than that of the liquid core. Therefore, at the inner wall, a portion of excitation light enters the wall while the rest is reflected within the liquid core. When the excitation light reaches the outer wall of the LCW, if the angle β is larger than the critical incident angle between the wall and the air, as the refractive index of the wall is higher than that of the surrounding air, TIR occurs. If α is small enough, all the excitation light is confined within the LCW. Simultaneously, as the direction of the emission light is isotropic, a large amount of emission light is able to exit the LCW and reach the detector. Therefore, the LCW performs the dual function of excitation guiding and emission filtering, in a single component, which is a key contributor to overall system size reduction.

**Figure 6:**
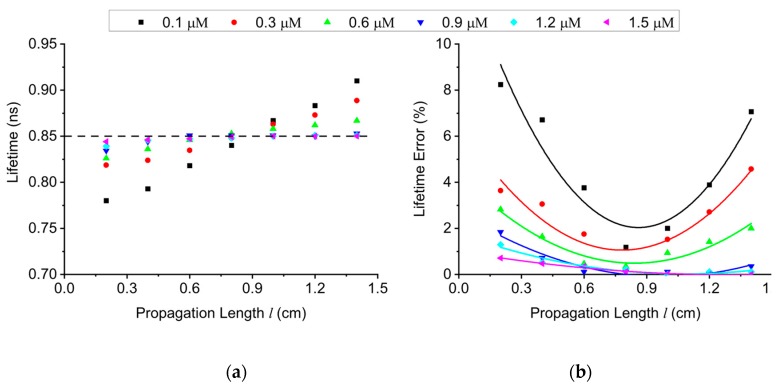
Monte Carlo simulation of extracted lifetime across V-carbazole concentrations, modeling time dispersion within the liquid core waveguide. (**a**) Lifetime extracted with different values of propagation length *l*, compared to the ideal value of 0.85 ns; (**b**) lifetime error at different propagation lengths, suggesting the optimal *l* is approximately 0.8 cm.

Although LCW offers significant potential for low-cost, portable microsystems, when excitation light travels within the LCW, partial reflections occur thereby decomposing the excitation into multiple split rays. As a result, a fluorescent molecule at one particular location is excited by different split rays, leading to temporal dispersion [24] and degradation in lifetime extraction accuracy. The fluorescence lifetime extraction accuracy is influenced by both the temporal dispersion [24] and the emission-to-excitation ratio [25]. The temporal dispersion needs to be smaller than the FWHM of the single photon detector transit time spread (TTS). Therefore, a small propagation length *l*, i.e., the length of the LCW, is desired [26]. On the other hand, based on Monte Carlo simulation, a small *l* reduces the emission-to-excitation ratio, thus degrading lifetime extraction accuracy. Therefore, the selection of *l* involves the tradeoff between low temporal dispersion and high emission-to-excitation ratio. In fact, the lower and upper boundaries of *l* are confined by the emission-to-excitation ratio and temporal dispersion, respectively. Given that the wall thickness and inner diameter of the employed LCW are 0.8 mm and 6.4 mm, respectively, the simulated lifetime across V-carbazole concentrations is shown in Figure 6a. Figure 6b depicts the corresponding lifetime error (*err*) calculated by

(1) $$err=\frac{LT_{S}-LT_{T}}{LT_{T}}\times 100\%$$

where $LT_{S}$ and $LT_{T}$ are measured lifetime and theoretical lifetime, respectively. Monte Carlo simulations suggest that the optimal value of propagation length is about 0.8 cm.

In practice, due to scattering, perfect excitation rejection cannot be achieved. For the highest level of performance, an emission filter can be optionally used. In addition, to prevent pile-up for the detector [27], as is typical for many TCSPC implementations, a neutral density (ND) filter can be inserted between the excitation source and the LCW to attenuate excitation power. In the proposed system, a FUJIFILM 10% thin-film ND filter is used.

### 4.2. Electronics Implementation

#### 4.2.1. Time-to-digital Conversion Module

Pico-second timing resolution is required from the time-to-digital conversion process. The Texas Instruments (TI) TDC7200 chip, an integrated time-to-digital converter (TDC) with least significant bit (LSB) timing resolution of 55 ps and an internal self-calibrated timer has been selected. The TDC has been configured to continuously compensate for drift and temperature over time.

#### 4.2.2. MCU

An MCU serves as the main controller for the TCSPC system, with which photon data from the TDC and user commands from a PC are handled. TI low-power MSP430F5529 has been employed as the MCU. Instead of transmitting the raw output data from the TDC directly to the PC, the conversion results are preprocessed by the MCU, including data reduction, to improve the overall data throughput of the system.

#### 4.2.3. User Interface

Top-level control of the proposed system is performed by a customized graphical user interface (GUI). A MATLAB script sends user controls via USB to the MCU to configure and operate the TCSPC hardware. After each measurement, the measured instrument response function (IRF) curve, the histogram of fluorescence decay, and the corresponding fluorescence lifetime are displayed for user convenience.

## 5. Results

### 5.1. Electronical Characteristics

**Figure 7:**
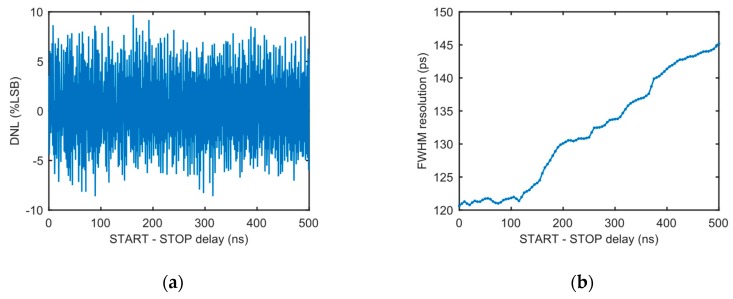
Measured (**a**) differential nonlinearity (DNL) and (**b**) full width at half maximum (FWHM) timing resolution of the time-to-digital conversion module in the proposed TCSPC system.

Two of the most important internal electrical characteristics of the TCSPC system, differential nonlinearity (DNL) and full width at half maximum (FWHM) timing resolution of the time-to-digital module, are experimentally measured and depicted in Figure 7. Within the 500 ns full-scale range (FSR), the DNL ranges between −8.5% and +9.7% of the LSB. The FWHM timing resolution of the time-to-digital module ranges from 121 to 145 ps. This level of electrical performance ensures the accuracy of subsequent lifetime extraction.

### 5.2. Validation of Emission Filtering

**Figure 8:**
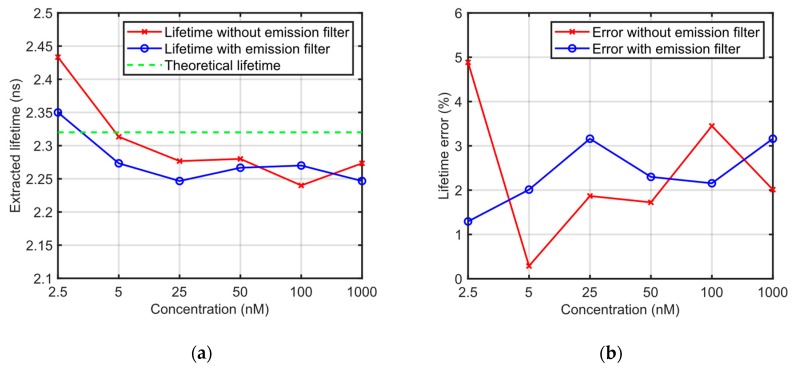
Characterization results of emission filtering performance of LCW. (**a**) Fluorescent lifetime of Coumarin 6 in dimethyl sulfoxide (DMSO) measured by the proposed TCSPC system with and without the emission filter, compared against the theoretical value of 2.32 ns; (**b**) measured lifetime error with and without the emission filter. Lifetime error of the TCSPC system is less than 5%, even without the employment of emission filter, indicating that most of the excitation light is rejected by the LCW.

Coumarin 6, a widely used fluorescent dye, is used to validate the emission filtering performance of LCW. The length of LCW is customized to its optimal value of 0.8 cm as discussed in Section 4.1.3. Different concentrations of Coumarin 6 in dimethyl sulfoxide (DMSO) are measured by the proposed TCSPC system with and without the emission filter, with fluorescence lifetime separately extracted using non-linear least square (NLLS) method. The measured lifetimes and errors are shown in Figure 8, indicating that most of the excitation light is rejected by the LCW, even without the employment of emission filter.

### 5.3. DNA Detection

**Figure 9:**
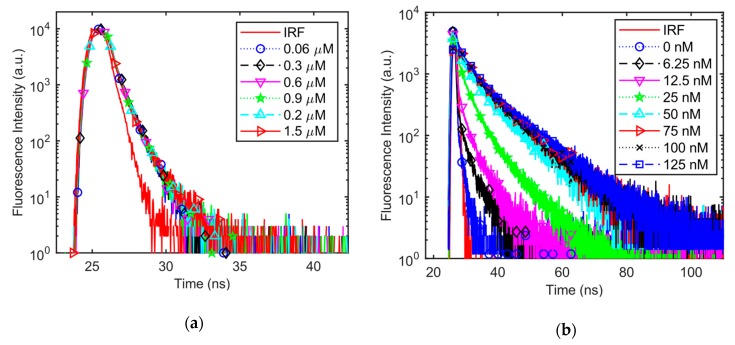

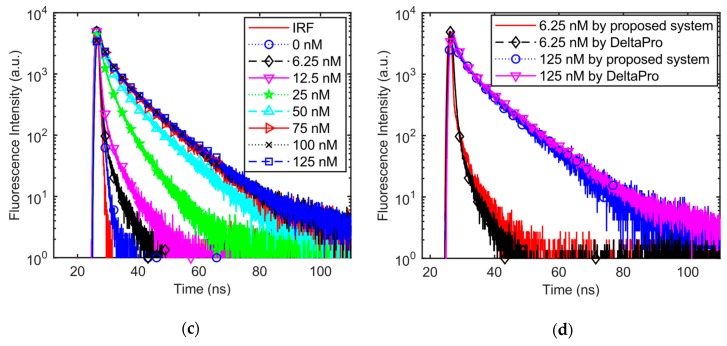
Measured instrument response function (IRF) and decays of the V-carbazole fluorophore with and without dsDNA. (**a**) IRF and the fluorescence decays of V-carbazole only (i.e., without DNA); (**b**) IRF and the fluorescence decays of 0.6 μM V-carbazole bound with DNA in various concentrations, measured by the proposed system; (**c**) IRF and the fluorescence decays of 0.6 μM V-carbazole bound to DNA, measured by DeltaPro (for comparison); (**d**) fluorescence decays of 0.6 μM V-carbazole bound to 6.25 nM and 125 nM DNA, measured by both systems.

The DNA detection capability of the proposed system is verified by fluorescence lifetime characterization of the V-carbazole based bioassay, with and without the target DNA, as shown in Figure 9. Figure 9a shows the measured IRF and the fluorescence decays of V-carbazole without the target DNA. Since V-carbazole molecules are in the OFF state, DNA concentration hardly influences the measured fluorescence lifetime. Figure 9b,c shows the IRF and the fluorescence decays of 0.6 μM V-carbazole bound with eight DNA concentrations (from 0 nM to 125 nM), measured by the proposed system and the Horiba DeltaPro (as a reference), respectively. Figure 9b,c shows that, as the DNA concentration increases, more DNA molecules are available to bind with the V-carbazole molecules. As more V-carbazole molecules are turned on, fluorescence lifetime is increased. In Figure 9d, the fluorescence decay results of 0.6 μM V-carbazole bound with 6.25 nM and 125 nM DNA are plotted for both the proposed TCSPC system and DeltaPro. Results from both systems are comparable, albeit the proposed TCSPC system is much more compact.

During each measurement of IRF and fluorescence decays, a sufficient amount of conversion data is needed for accurate fluorescence lifetime characterization. On the other hand, it is generally known that the emission-to-excitation ratio should be less than approximately 2% to avoid pile-up distortion, which degrades the lifetime measurement accuracy. Under these conditions, the proposed TCSPC system collects approximately 2 × 10^7^ samples within 2 min for each measurement, with a conversion speed up to 160 k samples per second (sps).

### 5.4. Fluorescence Lifetime Extraction

If the excitation process is of finite width (i.e., the IRF), and the background of both IRF and decay is small enough not to contribute significantly to the calculation of the barycenter, the mean fluorescence lifetime $\bar{\tau }$ can be calculated as the distance between the barycenter of the IRF and the barycenter of the decay curve [28]:

(2) $$\bar{\tau }=\frac{\int_{0}^{\infty }D\left(t\right)tdt}{\int_{0}^{\infty }D\left(t\right)dt}-\frac{\int_{0}^{\infty }IRF\left(t\right)tdt}{\int_{0}^{\infty }IRF\left(t\right)dt},$$

where *t* is the duration of the decay signal, *D(t)* and *IRF(t)* are the measured decay and IRF, respectively.

**Figure 10:**
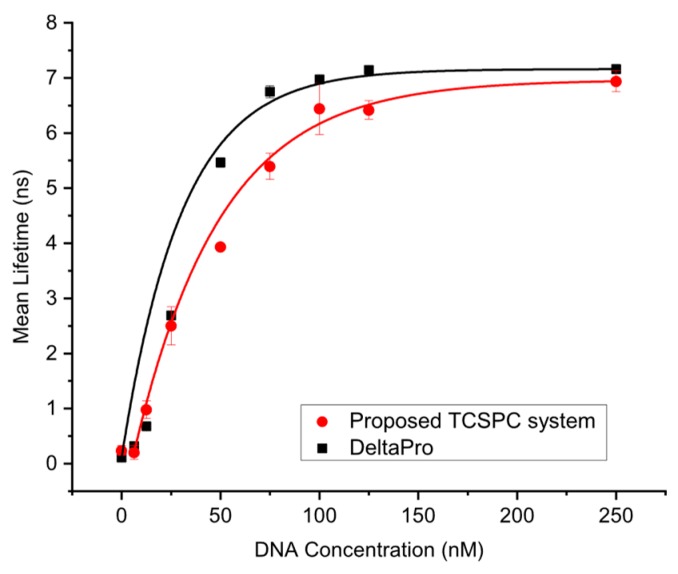
Mean and standard deviation of extracted fluorescence lifetimes across DNA concentrations, from both the proposed system and the DeltaPro.

The mean value and standard deviation of the fluorescence lifetime across DNA concentrations extracted using the proposed TCSPC system are depicted in Figure 10, and as a reference compared against the mean fluorescence lifetime extracted by the DeltaPro. The detection limit and dynamic range of the proposed TCSPC system are 6.25 nM and 6.8 ns, respectively. When the DNA concentration is larger than 50 nM, the mean fluorescence lifetime extracted using the proposed TCSPC system is slightly smaller than that of the DelraPro. This is because the detectors employed by DeltaPro and the proposed TCSPC system are PMT and SPAD, respectively. The active area of the SPAD is smaller than that of the PMT, thus a longer measurement time is needed to acquire enough data for lifetime extraction. However, prolonged excitation of fluorescent molecules leads to photobleaching, a photochemical alteration that the fluorescent molecules lose their capacity to fluoresce, resulting in the degradation in lifetime extraction accuracy. This is a limitation of using an SPAD. However, as SPAD technologies continue to improve rapidly, this performance gap is likely to close in the near future. Despite the above, the overall results show that the lifetime curves obtained from the proposed system is similar to that of the DeltaPro.

## 6. Discussion

In principle, detection efficiency changes with different DNA lengths, as different lengths cause variations of local environment, which may affect the lifetime of the V-carbazole. For clinical applications such as drug delivery, it is often suitable to use DNA with a length of 15 base pairs, which is the result of a tradeoff. On one hand, V-carbazole prefers to bind within the minor groove [21], shorter length of DNA lacking in minor groove reduces the binding probability and detection efficiency. On the other hand, longer DNA base pairs increase the size of the DNA nanomaterial, making the drug delivery process difficult to complete. Therefore, DNA with the length of 15 base pairs is experimentally used in this work, with a performance level suitable for application of using self-assembled DNA nanomaterials to deliver targeted drug to specific organelles such as mitochondria and cancer cells [22,29].

In this work, a low-cost TCSPC portable DNA analyzer by utilizing lifetime analysis technique and optimizing system composition is presented. As similar lifetime analysis techniques have demonstrated successful detection of mutation and mismatch of DNA and RNA [30,31,32,33,34], the feasibility of the proposed system for the detection of above bio-samples is worthy of further investigation.

## 7. Conclusions

In this paper, a portable, low-cost TCSPC system for DNA detection is presented. Optimized optical and electronic subsystems provide effective emission filtering and size reduction simultaneously, delivering good DNA detection and fluorescence lifetime extraction performance. With the dimension of 400 cm^3^, the proposed system is able to detect DNA with the length of 15 base pairs at the concentration of 6.25 nM. Experimental results from DNA-hybridized V-carbazole bioassay corroborate its applicability in clinical application environments such as targeted drug delivery, paving the way to a compact, integrated TCSPC technology for point-of-care DNA analysis.
